# Correlation and Agreement Between Parent‐Report and Self‐Report Food Allergy Quality of Life Questionnaires After Oral Immunotherapy

**DOI:** 10.1111/cea.70352

**Published:** 2026-05-25

**Authors:** Sophie A. Rosser, Melanie Lloyd, Rachel L. Peters, Rushani Wijesuriya, Paxton Loke, Sarah E. Ashley, Adriana Chebar Lozinsky, Mimi L. K. Tang

**Affiliations:** ^1^ Allergy Immunology Murdoch Children's Research Institute Parkville Victoria Australia; ^2^ Department of Paediatrics University of Melbourne Parkville Victoria Australia; ^3^ National Allergy Centre of Excellence (NACE) Parkville Victoria Australia; ^4^ Health Economics and Policy Evaluation Group Monash University Parkville Victoria Australia; ^5^ Population Allergy Murdoch Children's Research Institute Parkville Australia; ^6^ Centre for Food Allergy Research Murdoch Children's Research Institute Parkville Australia; ^7^ Clinical Epidemiology and Biostatistics Unit Murdoch Children's Research Institute Parkville Victoria Australia; ^8^ Department of Paediatrics Monash University Clayton Victoria Australia; ^9^ Monash Children's Hospital Monash Health Clayton Victoria Australia; ^10^ Allergy and Immunology The Royal Children's Hospital Parkville Victoria Australia

**Keywords:** food allergy, oral immunotherapy, paediatrics, quality of life

## Abstract

- We observed moderate correlation, but limited agreement, between parent‐ and self‐report FAQLQ instruments post‐treatment.
- FAQLQ instruments are not interchangeable, with score differences exceeding clinically acceptable thresholds.

**Abbreviations**
CCC: Lin's Concordance Correlation Coefficient
FAQLQ: Food Allergy Quality‐of‐Life Questionnaire
FAQLQ‐CF: Food Allergy Quality‐of‐Life Questionnaire–Child Form
FAQLQ‐PF: Food Allergy Quality‐of‐Life Questionnaire–Parent Form
FAQLQ‐TF: Food Allergy Quality‐of‐Life Questionnaire–Teenager Form
HRQL: Health‐related quality of life
LoA: Bland–Altman limits of agreement
OIT: oral immunotherapy
PPOIT‐003: Probiotic and Peanut oral immunotherapy‐003 trial
PEAT: Probiotic Egg Allergen Oral Immunotherapy for Treatment of Egg Allergy study
PrEMO: Probiotic and Egg or Milk Oral Immunotherapy study

To the Editor,

Food allergy has major impacts on health‐related quality of life (HRQL). The Food Allergy Quality‐of‐Life Questionnaires (FAQLQs) capture patient experiences of food allergy across distinct parent proxy‐report, and child (8–12 years) and teenager (13–17 years) self‐report perspectives, and are the recommended instruments for evaluation of HRQL in oral immunotherapy (OIT) studies [1]. However, correlation and agreement between parent‐ and self‐report FAQLQs have not been evaluated post‐treatment, and it remains unclear whether they can be used interchangeably as participants age during follow‐up.

We analysed FAQLQ total and subscale scores collected 12–24 months post treatment from a pooled sample of 86 parent–child and 14 parent‐teenager dyads enrolled in three trials evaluating the effectiveness of an 18‐month course of peanut, egg or milk OIT at inducing remission of food allergy.

Briefly, the PPOIT‐003/PPOIT‐003LT trial and follow‐on study (ACTRN12616000322437; HREC 35246/RGS2543) assessed peanut OIT with and without probiotic compared with placebo (randomised 2:2:1) in 201 children aged 1–10 years at study entry across Melbourne, Adelaide and Perth in Australia [2]. PEAT (ACTRN12619000480189; HREC 2019.082/2019/00029) evaluated egg OIT with probiotic versus placebo (randomised 1:1) in 41 participants aged 5–23 years at study entry in Melbourne, Australia and Singapore [3]. PrEMO (ACTRN12619000306112; HREC 48567/RCHM‐2019) was an open‐label study of egg or milk OIT with probiotic in 40 children aged 5–17 years at study entry in Melbourne, Australia [4]. Written informed consent was obtained from parents/guardians of the participants in all trials. Trial alignment, FAQLQ score calculation and participant selection details are available in the repository.

Correlation between parent‐ and self‐reported FAQLQ scores was assessed using Spearman's correlation coefficients (*r*_*s*_). Overall degree of agreement between FAQLQ scores was assessed with Lin's Concordance Correlation Coefficients (CCC) and scatterplots comparing the linear relationship between parent‐ and self‐reported scores to a line of perfect concordance (where theoretically, parent‐reported = self‐reported scores). Bland–Altman analysis was used to estimate the mean difference (MD) and 95% limits of agreement (LoA) between parent‐ and self‐reported scores. We considered an LoA width < 0.5 units between parent‐ and self‐reported scores as clinically acceptable for interchangeability, based on the established 0.5 unit minimum clinically important difference for the FAQLQ‐PF [5] and for 7‐point Likert scales measuring quality of life [6].

Moderate positive correlations were observed between parent‐ and child‐reported FAQLQ total scores (*r*_*s*_ = 0.63, 95% CI: 0.47–0.76), as well as parent‐ and teenager‐reported total scores (*r*_*s*_ = 0.80, 95% CI: 0.34–0.98), indicating moderate association between parent‐ and self‐report perspectives.

Moderate‐to‐strong agreement was observed between parent‐ and child‐reported FAQLQ total scores (CCC = 0.60, 95% CI: 0.46–0.72) (Figure 1A), and parent‐ and teenager‐reported total scores (CCC = 0.80, 95% CI: 0.50–0.93), though estimates were imprecise, particularly for teenagers (Figure 1B). When HRQL was generally good (low end of FAQLQ scale), self‐reported scores were slightly poorer (higher) than parent‐reported scores. Conversely, when HRQL was generally poor (high end of FAQLQ scale), self‐reported scores were lower (better) than parent‐reported scores. This pattern was descriptive and should not be interpreted as evidence of systematic differences between self‐ and parent‐reports.

**FIGURE 1:**
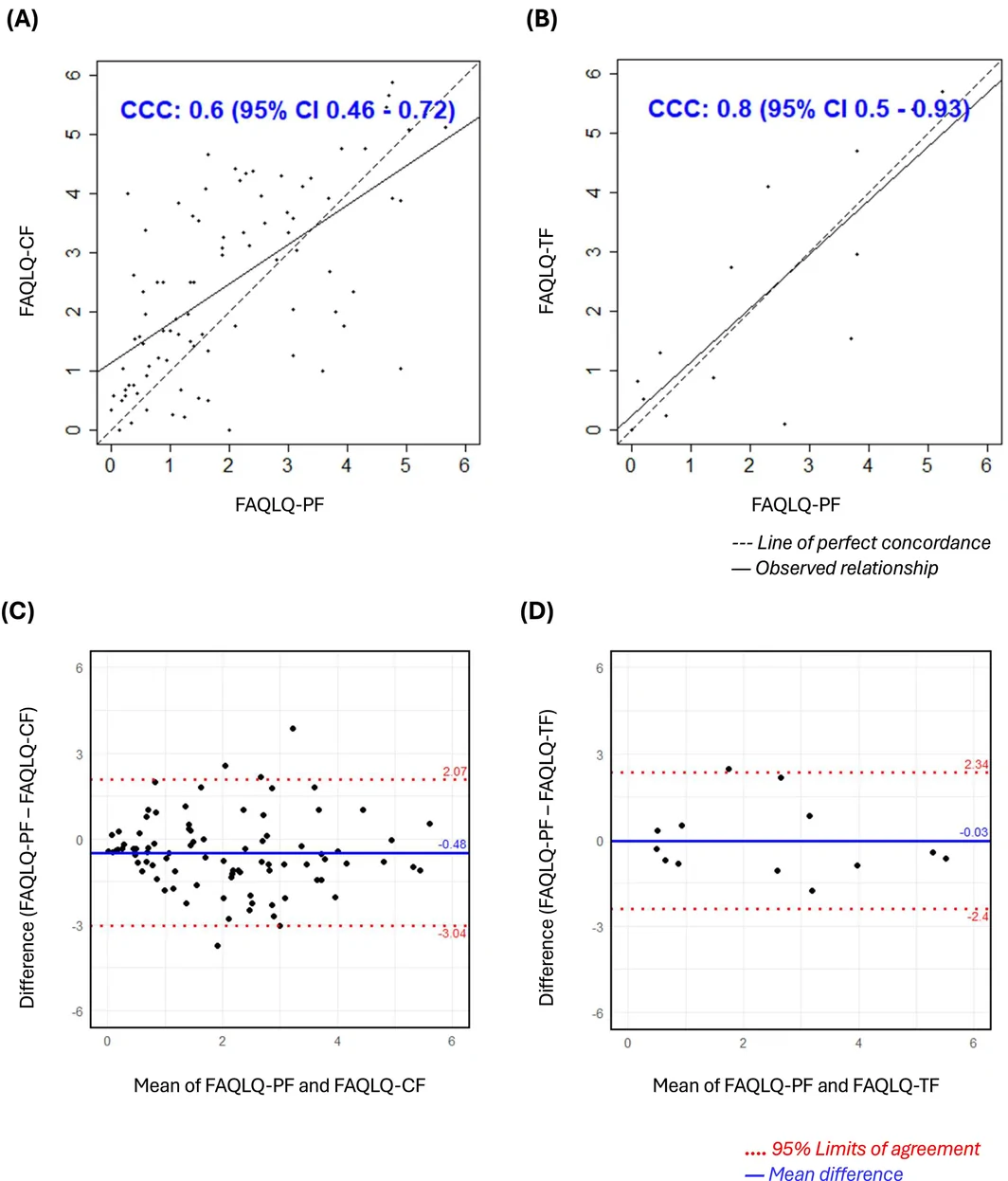
Agreement between parent‐ and self‐reported FAQLQ total scores. Lin's CCC plots comparing (A) the FAQLQ‐PF and FAQLQ‐CF (*N* = 86 dyads) and (B) the FAQLQ‐PF and FAQLQ‐TF (*N* = 14 dyads). Bland–Altman plots comparing (C) the FAQLQ‐PF and FAQLQ‐CF (*N* = 86 dyads) and (D) FAQLQ‐PF and FAQLQ‐TF (*N* = 14 dyads). The FAQLQ instruments measure HRQL on the same scale of 0–6 units, with lower scores indicating better HRQL. CI, confidence interval; CCC, Lin's concordance correlation coefficient; FAQLQ‐CF, Food Allergy Quality of Life Questionnaire—Child Form; FAQLQ‐PF, Food Allergy Quality of Life Questionnaire—Parent Form; FAQLQ‐TF, Food Allergy Quality of Life Questionnaire—Teenager Form.

Bland–Altman analysis indicated that parent‐reported total FAQLQ scores were on average 0.48 units lower (better) than child‐reported total scores with very wide LoA ranging from −3.04 to 2.07 units (Figure 1C). These scores were not sufficiently similar to be considered interchangeable, with variability far exceeding the clinically meaningful threshold.

A far smaller mean difference of −0.03 units and less variability were observed between parent‐reported and teenager‐reported total FAQLQ scores, though the LoA remained wide (−2.40 to 2.34) and again refuted interchangeability (Figure 1D).

Findings were consistent in comparisons of FAQLQ subscale scores (in repository).

Despite moderate correlation, disagreement between parent‐ and self‐reported scores was considerable and exceeded clinically acceptable limits, precluding their use as interchangeable measures.

This is broadly consistent with findings from previous non‐interventional studies [7, 8]. However, whereas prior work reported consistently better HRQL by parent‐report than child‐report, we demonstrated a potentially more complex pattern of association between instruments post‐OIT, with parent‐reported scores suggesting greater benefit when HRQL was good and greater burden when HRQL was poor. This highlights that the direction and magnitude of differences between reporting perspectives may vary across the FAQLQ scale.

A previous study of parent‐ and child‐reported scores during OIT and short‐term follow‐up demonstrated HRQL improvement across both parent‐ and child‐reported FAQLQs, with consistently lower (better) scores reported by parents [9]. While correlation and agreement between FAQLQ perspectives were not formally assessed in that study, it provides helpful context to our observation that parent‐ and self‐reported FAQLQs are well correlated but not interchangeable. The parent‐ and self‐report FAQLQs may inherently measure HRQL differently, such that absolute scores obtained between instruments differ, not the conclusions reached.

Nonetheless, the lack of interchangeability between FAQLQs has important implications for longitudinal interventional studies. Switching from parent‐ to self‐report FAQLQs during longitudinal follow‐up may introduce artefactual changes in HRQL that reflect reporting perspective rather than true clinical change. These findings support the importance of using a consistent reporting instrument across time points or incorporating parallel parent‐ and self‐reports during periods of transition.

A key limitation in this study is potential selection bias. The opportunistic nature of our analysis, small sample size, predominance of peanut allergy and potential for enrichment of highly engaged families in longitudinal assessments may have biased estimates towards greater alignment between reporters and inflated the magnitude of correlation and agreement estimates. Despite these limitations, few OIT trials have assessed HRQL from multiple perspectives in long‐term follow‐up, and this analysis represents the most comprehensive dataset currently available. Our findings provide important guidance for HRQL measurement in paediatric food immunotherapy trials and highlight the need for standardised approaches to longitudinal assessment.

## Author Contributions

S.A.R., M.L., R.L.P., R.W. and M.L.K.T. conceived this study and designed the methodology. S.A.R. conducted the analysis and drafted the preliminary version of the manuscript. All authors assisted with interpretation of the results and reviewed and critically appraised the manuscript before submission.

## Funding

S.A is supported by the Commonwealth through an Australian Government Research Training Program Scholarship [DOI: https://doi.org/10.82133/C42F‐K220] and by a PhD scholarship from the Australian Government funded National Allergy Centre of Excellence (NACE), hosted by the Murdoch Children's Research Institute (MCRI), and their work was supported by the Victorian Government's Operational Infrastructure Support Program. The PPOIT‐003 randomized trial is an investigator‐initiated study funded by National Health and Medical Research Council Australia Project (Grant 115423). PEAT is an investigator‐initiated study funded by a National Health and Medical Research Council (NHMRC) grant—Australia, Project Grant. PrEMO was jointly funded by Prota Therapeutics and the Murdoch Children's Research Institute.

## Conflicts of Interest

M.L.K. Tang declares consultant fees from Pfizer and CSL Seqirus; is a member of the Medical Advisory Board of Allergy & Anaphylaxis Australia; is a member of the Board of Directors of Asia Pacific Association of Allergy Asthma and Clinical Immunology, AllergyPal and Prota Therapeutics; is a member of expert committees of the American Academy of Allergy, Asthma & Immunology, Asia Pacific Association of Allergy Asthma and Clinical Immunology, Australasian Society of Clinical Immunology and Allergy, and World Allergy Organization. M.L. reports consultant fees from the National Allergy Centre of Excellence, Australia. R.L.P. has received research funding from the National Health and Medical Research Council (NHMRC), National Peanut Board, Asthma Australia, Thoracic Society of Australia and New Zealand (TSANZ), and Allergy and Immunology Foundation of Australasia (AIFA); a prize from the Stallergenes Greer Foundation; and speaker fees from Thermo Fisher Scientific, all paid to their institution outside of the submitted work. P.L. reports consultant fees from SPRIM Consulting, Prota Therapeutics and an institutional grant from Siolta Therapeutics. S.E.A. has received research funding from the NHMRC, AIFA, Ramaciotti Foundation, Biostime Institute of Nutrition and Care and consultant fees from the National Allergy Centre of Excellence, Australia, all paid to their institution and outside of the submitted work. The other authors declare no relevant conflicts of interest for this work.

## Data Availability

Additional methodology and summary data related to the results of this letter are available in the repository: https://osf.io/752tm/overview?view_only=f3ae0a7353024bb1828ab95062daab0b
